# Complete Genome Sequence of *Streptomyces* sp. Strain CCM_MD2014, Isolated from Topsoil in Woods Hole, Massachusetts

**DOI:** 10.1128/genomeA.01506-15

**Published:** 2015-12-31

**Authors:** Richard M. Mariita, Srijak Bhatnagar, Kurt Hanselmann, Mohammad J. Hossain, Jonas Korlach, Matthew Boitano, Richard J. Roberts, Mark R. Liles, Anthony G. Moss, Jared R. Leadbetter, Dianne K. Newman, Scott C. Dawson

**Affiliations:** a Department of Biological Sciences, Auburn University, Alabama, USA; b Microbiology Graduate Group, University of California Davis, Davis, California, USA; c Department of Earth Sciences, ETH Zurich, Zürich, Switzerland; d Pacific Biosciences, Menlo Park, California, USA; e New England BioLabs, Ipswich, Massachusetts, USA; f Linde Center for Global Environmental Science, California Institute of Technology, Pasadena, California, USA; g Division of Biology and Biological Engineering, California Institute of Technology, Pasadena, California, USA; h Howard Hughes Medical Institute, Pasadena, California, USA; i Division of Geological and Planetary Sciences, California Institute of Technology, Pasadena, California, USA; j Department of Microbiology and Molecular Genetics, University of California Davis, Davis, California, USA

## Abstract

Here, we present the complete genome sequence of *Streptomyces* sp. strain CCM_MD2014 (phylum *Actinobacteria*), isolated from surface soil in Woods Hole, MA. Its single linear chromosome of 8,274,043 bp in length has a 72.13% G+C content and contains 6,948 coding sequences.

## GENOME ANNOUNCEMENT

The genus *Streptomyces* belongs to the phylum *Actinobacteria*, the members of which are Gram-positive bacteria that are ubiquitous in soils and typically have a genome with a high G+C content. They are known for their capacity for secondary metabolite synthesis and expression of novel enzymes (1). The strain in this study, *Streptomyces* sp. CCM_MD2014, was cocultured with *Curtobacterium* sp. strain MR_MD2014 from topsoil around a rusty fire hydrant in Woods Hole, MA (41°31′44.65″N 70°40′21.5″W) on 7 July 2014, using isolation protocols modified from those of El-Nakeeb and Lechevalier (2). This organism was cultivated as part of the 2014 Microbial Diversity Summer Program at the Marine Biological Laboratory in Woods Hole, MA.

DNA was extracted from the coculture using the Promega Wizard genomic DNA purification kit with 1 h of lysozyme digestion. The DNA was quantified using the Promega QuantiFluor double-stranded DNA (dsDNA) system and then size selected for a minimum length of 4 kb. Size-selected DNA was sequenced on a Pacific Biosciences RSII sequencing platform with P5C3 chemistry. The sequenced fragments were assembled using HGAP3 on the SMRT Portal (3). The final assembled genome consisted of a single linear chromosome that was 8,274,043 bp long, with a 72.13% G+C content and sequencing coverage of 89×.

The genome was annotated using NCBI’s Prokaryotic Genome Annotation Pipeline version 2.8 (rev. 449627) (4, 5). There were 6,948 coding sequences (CDSs), 6 rRNA operons, and 68 tRNA genes. Neither Clustered Regularly Interspaced Short Palindromic Repeats (CRISPRs) nor prophages were detected in the genome by CRISPRFinder (6) and PHAST (7), respectively. The antiSMASH pipeline (8) predicted 52 secondary metabolite biosynthetic genetic clusters in the genome, including genes for lantipeptides, terpenes, siderophores, polyketide synthases type I and II, bacteriocins, and nonribosomal peptide synthase genes. REBASE (9) identified 13 candidate methylase genes, and the following methylated motifs were found using Pacific Biosciences SMRT Portal analysis: A^m6^AGNNNNNNNTCCG, CATCC^m6^AG, CC^m6^AGNNNGTCG, GACGA^m6^AC, and GCCGGC (see organism number 13448 on REBASE website for more details). A multilocus phylogenetic analysis using *rpoB*, *tryB*, *recA*, *gyrB*, and *atpD* and average nucleotide identity (ANI) (10) of 93.8% indicated that Streptomyces coelicoflavus ZGO656 is the closest sequenced genome. A concatenated alignment of these genes was used for phylogenetic reconstruction.

### Nucleotide sequence accession number.

The complete genome sequence of *Streptomyces* sp. strain CCM_MD2014 is available through GenBank under the accession number CP009754.
